# Quantitative comparison of novel GCaMP-type genetically encoded Ca^2+^ indicators in mammalian neurons

**DOI:** 10.3389/fncel.2012.00041

**Published:** 2012-10-08

**Authors:** Yoshiyuki Yamada, Katsuhiko Mikoshiba

**Affiliations:** ^1^Laboratory for Developmental Neurobiology, Brain Science Institute, RIKENWako-shi, Saitama, Japan; ^2^Central Institute for Experimental AnimalsKawasaki-shi, Kanagawa, Japan

**Keywords:** genetically encoded Ca^2+^ indicators, GCaMP, GECO, two-photon imaging, patch-clamp recording, cortical pyramidal cell, *in utero* electroporation, acute brain slice

## Abstract

New variants of GCaMP-type genetically encoded Ca^2+^ indicators (GECIs) have been continuously developed and heavily used in many areas of biology including neuroscience. The latest subfamily called “GECOs” were developed with *in vitro* high-throughput screening, and shown to have novel spectral properties and/or improved fluorescent responses over their ancestor GCaMP3. The most critical parameter in evaluating performance in neurons, however, remains uncharacterized: the relationship between the GECI responses and the number of action potentials (APs). Here we analyzed the GECI responses to APs in cortical pyramidal cells of mouse acute brain slices. Unexpectedly, we found that none of the GECOs exhibited any improved performance over GCaMP3. Our results imply that careful validation is required for the accurate prediction of the actual performance of GECIs in mammalian neurons. We propose that appropriate guidelines for evaluating their efficacy should be established for the benefit of research community, given the rapidly expanding use of GECIs in neuroscience.

## Introduction

Genetically encoded Ca^2+^ indicators (GECIs; for review: Mank and Griesbeck, 2008; Tian et al., 2012), or Ca^2+^-sensitive fluorescent proteins, are regarded as promising tools for many areas of biology including neuroscience. Since GECIs can in principle be stably expressed in a targeted type of cells, they emerged as critically important tools for analyzing long-term changes of *in vivo* multi-neuronal activity during learning, development and disease (Mank et al., 2008; Huber et al., 2012).

Among several types of GECIs, GCaMP family (Nakai et al., 2001) has attracted intense attention in the field. It consists of circularly-permutated GFP, calmodulin (CaM), and M13 (Ca^2+^/CaM-binding peptide derived from skeletal muscle myosin light chain kinase), and changes its fluorescence intensity in response to [Ca^2+^]_i_ changes. The first prototypes suffered from poor expression at mammalian physiological temperature (Nakai et al., 2001; Ohkura et al., 2005), but this problem was overcome by subsequent mutagenesis, resulting in GCaMP2 (Tallini et al., 2006). GCaMP2 was successfully used to monitor activation of cerebellar parallel fibers (Díez-García et al., 2005, 2007) and vomeronasal neurons (He et al., 2008), yet it turned out to be mostly insensitive to [Ca^2+^]_i_ changes caused by single or small number of action potentials (APs) (Mao et al., 2008). Taking advantage of the crystal structure information described (Wang et al., 2008; Akerboom et al., 2009), GCaMP2 was mutagenized into GCaMP3 to show higher baseline fluorescence and larger dynamic range (Tian et al., 2009). Although GCaMP3 has been widely used for recording *in vivo* activity of mammalian neurons (Dombeck et al., 2010; Huber et al., 2012; Keller et al., 2012), its detection reliability of single APs is still relatively low under physiological conditions (Tian et al., 2009; Yamada et al., 2011). This issue makes it difficult to relate the fluorescent responses with neuronal activity, and thus should be overcome in the next generation of GCaMP.

The most recent subfamily of GCaMP was developed with random mutagenesis of GCaMP3 accompanied by high-throughput *in vitro* screening (Zhao et al., 2011). The best variants with improved performance and/or novel spectral properties were efficiently selected from >10^5^ clones, and termed genetically encoded Ca^2+^ indicators for optical imaging (GECOs). The superior functionality of GECOs was confirmed by Ca^2+^ titration with purified proteins and live imaging of cancer cell line. All of the three variants of green GECOs (G-GECOs) with different dissociation constants (*K*_*d*_) for Ca^2+^ (G-GECO1.0, 750 nM; G-GECO1.1, 620 nM; and G-GECO1.2, 1150 nM) showed twice as large dynamic range as GCaMP3 (*K*_*d*_: 540 nM). Red-shifted GECO (R-GECO1; *K*_*d*_: 480 nM) showed dynamic range similar to GCaMP3. Blue-green emission ratiometric GECO (GEM-GECO1; *K*_*d*_: 340 nM) showed the largest dynamic range, 6–9-fold larger than GCaMP3. Furthermore, G-GECOs were shown to be superior to, and R-GECO1 comparable to, GCaMP3 in detecting spontaneous activity of cultured rat hippocampal neurons, and GEM-GECO1 was shown to be functional in sensory neurons of *C. elegans*.

Nevertheless, the most critical parameter of GECOs for evaluating their performance in neurons remains poorly characterized: the relationship between fluorescent responses and the number of APs. This leaves open the possibility that the GECO-expressing neurons happened to be more actively firing than GCaMP3-expressing neurons, resulting in apparently improved performance. In addition, most data in the original study were acquired in a culture system, where GECIs sometimes show larger responses compared to non-culture or *in vivo* systems (Tian et al., 2009). It therefore remains unclear whether GECOs can indeed show improved performance in mammalian neurons.

To address these issues, we analyzed the GECI responses to APs in cortical pyramidal cells of mouse acute brain slices by simultaneous two-photon imaging and patch-clamp recording, and investigated whether GECOs would indeed show better responses than their ancestor GCaMP3.

## Materials and methods

All experimental procedures were performed in accordance with the guidelines of the Animal Experiment Committee of the RIKEN Brain Science Institute.

### *In utero* electroporation

cDNAs encoding GCaMP3, G-GECO1.0, G-GECO1.1, G-GECO1.2, R-GECO1, and GEM-GECO1 were subcloned into a plasmid vector carrying the cytomegalovirus enhancer and β-actin (CAG) promoter, woodchuck hepatitis virus post-transcriptional regulatory element (WPRE) and bovine growth hormone (BGH) polyadenylation signal (Gray et al., 2006). To facilitate identification of mice or cells expressing GECIs, tdTomato (Shaner et al., 2004) was co-expressed with GCaMP3 and G-GECOs, and EGFP with R-GECO1 by internal ribosome entry site (IRES). All constructs were verified by DNA sequencing. *In utero* electroporation was performed as previously described (Saito, 2006; Shimogori and Ogawa, 2008). Briefly, embryonic day (E) 15 timed-pregnant ICR mice (Japan SLC) were anesthetized by intraperitoneal injection of sodium pentobarbital (approximately 50 mg/kg) and uterus horns were exposed on a heating pad (BWT-100, BRC). Approximately 1 μl of purified plasmid solution (1 μg/μl in phosphate buffer solution (PBS), with 0.02% Fast Green) was pressure-injected (IM-300, Narishige) into the lateral ventricle of embryos, and five electrical pulses (45 V, 50 ms duration at 1 Hz) were delivered through the uterine wall by a tweezer-type electrode (CUY650-P5, NEPA GENE) connected to an electroporator (CUY21-EDIT, NEPA GENE). After electroporation, the embryos were carefully replaced into the abdominal cavity, and the muscle and skin were sutured.

### Electrophysiology and two-photon imaging in acute brain slice

Parasagittal cortical slices (300 μm) were prepared using a vibratome (VT1000S, Leica) from electroporated mice on postnatal day (P) 14–25, as described previously (Davie et al., 2006). Brain dissection and slice preparation were performed in ice-cold cutting solution containing 87 mM NaCl, 75 mM sucrose, 2.5 mM KCl, 0.5 mM CaCl_2_, 7 mM MgCl_2_, 1.25 mM NaH_2_PO_4_, 25 mM NaHCO_3_, 10 mM _D_-glucose (320–330 mOsm/kg) and transferred to artificial cerebral spinal fluid (ACSF) containing 125 mM NaCl, 2.5 mM KCl, 2 mM CaCl_2_, and 1 mM MgCl_2_, 1.25 mM NaH_2_PO_4_, 25 mM NaHCO_3_, 25 mM _D_-glucose (310–320 mmol/kg), incubated at 34°C for 30–60 min and preserved at room temperature until use. Both cutting solution and ACSF were saturated with carbogen. Slices were perfused with ACSF warmed up to 33 ± 2°C by an in-line heater (TC-324B, Warner Instruments) at approximately 2 ml/min. GECI-expressing cortical layer 2/3 pyramidal cells were identified by epifluorescence and targeted for whole-cell patch-clamp recording under infra-red differential interference contrast microscopy (IR-DIC; Olympus). Electrophysiological signals were low-pass filtered at 3–10 kHz by 4-pole Bessel filter and acquired at 20–50 kHz using MultiClamp 700B (Molecular Devices) connected to ITC-16, ITC-18 (Instrutech) or Digidata 1440 (Molecular Devices) controlled by AxoGraphX (AxoGraph Scientific) or pClamp10 (Molecular Devices). Boroscilicate glass pipettes (4–7 MΩ) were filled with the internal solution containing 140 mM K-gluconate, 4 mM NaCl, 10 mM HEPES, 4 mM Mg-ATP, 0.3 mM Na-GTP and 5 mM Na_2_-phosphocreatine (pH 7.3 titrated with KOH, 285–295 mmol/kg). In experiments using Oregon Green 488 BAPTA-1 (OGB-1; Invitrogen), slices were prepared from non-electroporated mice, and 20 μM OGB-1 and 25 μM Alexa Fluor 594 (Invitrogen) was added to the pipette solution. APs were evoked by brief somatic current pulses (1–3 nA, 2 ms) delivered through recording patch pipettes.

For experiments with GECIs, image acquisition began typically after 2 min of break-in and terminated within 30 min given washout of GECIs (Pologruto et al., 2004; Mao et al., 2008). For experiments with OGB-1, image acquisition began after 15 min of break-in for equilibration of the dyes.

Fluorescent signals were acquired in line-scan mode (approximately 200 Hz) with an upright two-photon laser-scanning microscope (BX-61WI with FV300 or FV1000-MPE, Olympus) equipped with a 60× water-immersion objective (LUMPlan Fl/IR NA 0.90, Olympus). Imaging was performed across the proximal apical dendritic segments (<30 μm from the base) as described previously (Pologruto et al., 2004; Mao et al., 2008; Tian et al., 2009; Yamada et al., 2011). The Ti:sapphire laser (Maitai VF-TIM or Maitai DeepSee, Spectra-Physics) was tuned to 920 nm for GCaMP3 and G-GECOs, 990 nm for R-GECO1, 780 nm for GEM-GECO1 or 800 nm for OGB-1. Emitted fluorescence was short-pass filtered (650 or 690 nm, Olympus), split with a dichroic mirror, band-pass filtered and detected by photomultipliers (R3896, Hamamatsu). Details of optical filters are provided in Table 1.

**Table 1:** **Summary of optical filters (all from Olympus)**.

**GECIs**	**Optical filters**
GCaMP3, G-GECOs, OGB-1	DM: 570 nm
	Em: 495–540 nm
R-GECO1	DM: 570 nm
	Em: 575–630 nm
GEM-GECO1	DM: 485 nm
	Em: 420–460 nm (“blue”), 495–540 nm (“green”)

The electrophysiological recording and 2-photon imaging were synchronized by a trigger pulse generated upon laser scanning.

### Data analysis

After subtraction of dark noise on the photomultipliers, the mean baseline fluorescence (*F*_0_, GCaMP3, G-GECOs, R-GECO1, and OGB-1) or the mean baseline ratio of blue to green fluorescence (*R*_0_, GEM-GECO1) was calculated as the mean fluorescence or the mean ratio, respectively, of the approximately 1 s window immediately before stimulus onset (baseline period). Subsequently, the fractional change of the fluorescence (Δ*F*/*F*_0_, GCaMP3, G-GECOs, R-GECO1, and OGB-1) or the fractional change of the ratio (Δ*R*/*R*_0_, GEM-GECO1) was calculated. To facilitate comparison across GECIs with different baseline noise level, the signal-to-noise ratio (SNR) was calculated as Δ*F*/*F*_0_ or Δ*R*/*R*_0_ divided by the baseline standard deviation. Peak SNR was calculated from SNR trace filtered with a 35 ms moving window and defined as the maximum value between the stimulus onset and 500 ms after the stimulus cessation. SNR was calculated from individual trials and averaged over 3 trials for each stimulus condition. Responses were judged to be suprathreshold when SNR exceeds 2. Half rise time and half decay time were calculated from the 3-trial-averaged and filtered traces for 10 APs only when responses were suprathreshold. Statistical difference was assessed using Kruskal–Wallis test (*p* = 0.05) followed by Dunn's *post-hoc* test to compare GCaMP3 and one of the GECOs individually, unless otherwise noted. Data analysis was performed with AxoGraphX, Igor Pro 6 (WaveMetrics), NeuroMatic (http://www.neuromatic.thinkrandom.com/), Fluoview (Olympus), ImageJ (US National Institutes of Health), Excel (Microsoft) and GraphPad Prism4 (GraphPad software). All values are presented as mean ± standard deviation and error bars show standard deviation.

## Results

### The expression pattern of GCaMP3 and GECOs

**Figure 1:**
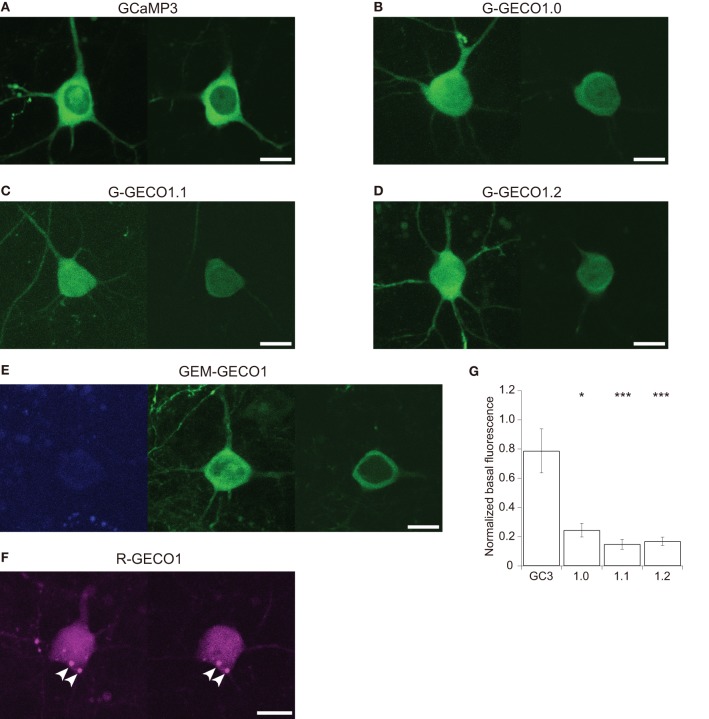
**Expression patterns of GCaMP3 and GECOs in cortical layer 2/3 pyramidal cells. (A–D)** Representative expression patterns of GCaMP3 **(A)** G-GECO1.0 **(B)** G-GECO1.1 **(C)** G-GECO1.2 **(D)**. Maximum intensity projection images (left) and single z-section images containing the nucleus (right) of GECIs are shown. **(E)** A representative expression pattern of GEM-GECO1. A maximum intensity projection image of the blue channel (left) and a maximum intensity projection image of the green channel (center) and a single z-section of the green channel containing the nucleus (right) are shown. **(F)** A representative expression pattern of R-GECO1. A maximum intensity projection image (left) and a single z-section containing the nucleus (right) of R-GECO1 are shown. Punctate structures are indicated by arrowheads. Image contrast was adjusted to clarify the presence or absence of nuclear fluorescence. Scale bar represents 10 μm. **(G)** The mean baseline fluorescence intensity of GCaMP3 and G-GECOs normalized by the fluorescence intensity of co-expressed tdTomato (*n* = 13 for GCaMP3, *n* = 10 for G-GECO1.0 and G-GECO1.1, and *n* = 9 for G-GECO1.2). Significant difference in Dunn's *post-hoc* test comparing GCaMP3-expressing cells and GECO-expressing cells: ^*^p < 0.05, ^***^p < 0.001.

We expressed GCaMP3 and GECOs in mouse cortical layer 2/3 pyramidal cells by *in utero* electroporation. GCaMP3, G-GECOs and GEM-GECO1 were expressed normally in the cytosol, with majority of cells lacking fluorescence in the nucleus (Figures 1A–E). In contrast, R-GECO1 was typically expressed in the nucleus as well as the cytosol, where it showed punctate structures (Figure 1F). This is reminiscent of other coral-derived fluorescence proteins that are resistant to proteolysis in acidic organella (Hirrlinger et al., 2005; Katayama et al., 2008, 2011; Perron et al., 2009).

G-GECOs had much lower basal fluorescence than GCaMP3 (Figure 1G; normalized fluorescence intensity of GCaMP3, 0.79 ± 0.15, *n* = 13; G-GECO1.0, 0.25 ± 0.05, *n* = 10, *p* < 0.05; G-GECO1.1, 0.15 ± 0.03, *n* = 10, *p* < 0.001; G-GECO1.2, 0.17 ± 0.03, *n* = 9, *p* < 0.001), which could be a significant disadvantage for *in vivo* application.

### The performance of GCaMP3 and GECOs in cortical layer 2/3 pyramidal cells

We characterized the performance of GECIs expressed in the cortical layer 2/3 pyramidal cells by simultaneous 2-photon imaging and whole-cell patch-clamp recording in acute brain slice preparations. Overall, the expression of GECIs did not have significant effects on the electrophysiological properties of pyramidal cells, except G-GECO1.0 and GEM-GECO1, the expression of which seemed to result in higher threshold and broader half width of APs (Table 2).

**Table 2:** **Electrophysiological properties of layer 2/3 pyramidal cells expressing GCaMP3 and GECOs**.

**Ephys**	**GC3 (*n* = 13)**	**1.0 (*n* = 10)**	**1.1 (*n* = 10)**	**1.2 (*n* = 9)**	**R (*n* = 9)**	**GEM (*n* = 9)**	**no exp (*n* = 11)**
*V*_m_ ^a^	−80 ± 5.8	−81 ± 4.8	−82 ± 5.6	−82 ± 4.1	−78 ± 2.1	−80 ± 3.8	−82 ± 8.7
*R*_m_ ^b^	135 ± 32	107 ± 44	124 ± 54	134 ± 66	128 ± 25	113 ± 31	139 ± 42
AP amplitude ^c^	100 ± 6.8	98 ± 9.2	103 ± 8.1	101 ± 8.0	105 ± 7.1	100 ± 4.9	107 ± 19
AP threshold ^d^	−43 ± 5.5	−41 ± 5.1^*^	−45 ± 3.6	−42 ± 3.7	−43 ± 7.7	−37 ± 5.5^***^	−47 ± 7.4
AP half width ^e^	2.0 ± 0.8	2.8 ± 1.1^**^	2.0 ± 0.6	2.1 ± 0.6	2.2 ± 1.4	4.2 ± 2.7^***^	1.7 ± 0.5

All values are corrected for liquid junction potential (12 mV). Significant difference in Dunn's post-hoc test comparing GECI-expressing cells and wild-type cells:

* *p* < 0.05;

** *p* < 0.01;

*** *p* < 0.001. *GC3, GCaMP3; 1.0, G-GECO1.0; 1.1, G-GECO1.1; 1.2, G-GECO1.2; R, R-GECO1; GEM, GEM-GECO1; no exp, cells from wild-type animals without OGB-1.*

a Resting membrane potential (mV).

b Input resistance (MΩ).

c Amplitude of action potential measured from the resting membrane potential (mV).

d Threshold voltage for action potential generation defined as the point where the first temporal derivative of the voltage first exceeds 50 mV/ms (mV).

e Full width of action potential measured at half the amplitude (ms).

**Figure 2:**
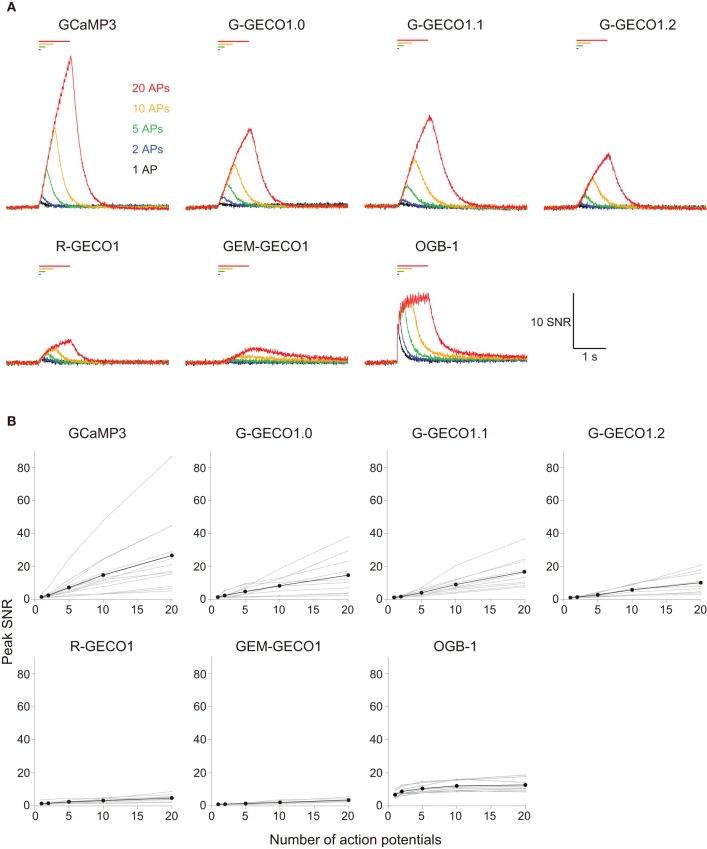
**Action potential-evoked responses of GCaMP3, GECOs, and OGB-1 in cortical layer 2/3 pyramidal cells. (A)** SNR traces in response to 1 (black), 2 (blue), 5 (green), 10 (orange) and 20 (red) action potentials (APs) evoked at 20 Hz. Each trace is the mean across cells (*n* = 13 for GCaMP3, *n* = 10 for G-GECO1.0 and G-GECO1.1, *n* = 9 for G-GECO1.2, R-GECO1 and GEM-GECO1, and *n* = 8 for OGB-1). **(B)** Peak SNR of GECI responses plotted against the number of APs evoked at 20 Hz. Gray represents data from individual cells and black the mean across cells.

**Figure 3:**
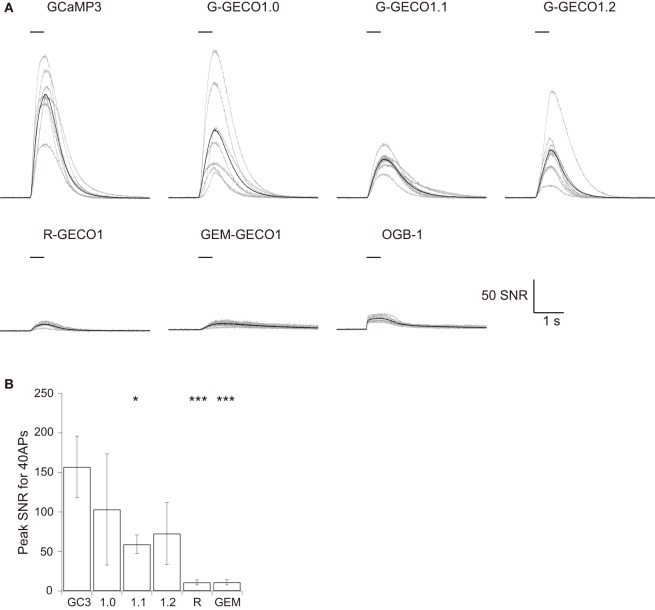
**Responses of GCaMP3, GECOs, and OGB-1 to 40 APs evoked at 83 Hz. (A)** SNR traces in response to 40 APs evoked at 83 Hz. Gray traces are from individual cells and black traces represent the mean across cells (*n* = 8 for GCaMP3, R-GECO1, and OGB-1, *n* = 7 for G-GECO1.0, and *n* = 9 for G-GECO1.1, G-GECO1.2 and GEM-GECO1). **(B)** The mean peak SNR for 40 APs evoked at 83 Hz. Significant difference in Dunn's *post-hoc* test comparing GCaMP3-expressing cells and GECO-expressing cells: ^*^p < 0.05, ^***^p < 0.001.

**Figure 4:**
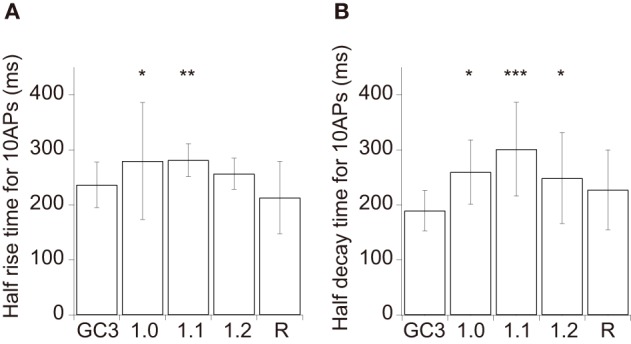
**Kinetics of GCaMP3 and GECOs in cortical layer 2/3 pyramidal cells. (A,B)** The mean half rise time **(A)** and mean half decay time **(B)** of smoothed trial-averaged traces in response to 10 APs at 20 Hz (*n* = 13 for GCaMP3, *n* = 9 for G-GECO1.0 and G-GECO1.2, *n* = 10 for G-GECO1.1, and *n* = 7 for R-GECO1). GEM-GECO1 was excluded from analysis due to the small number of cells showing suprathreshold responses (*n* = 4). Significant difference in Dunn's *post-hoc* test comparing GCaMP3-expressing cells and GECO-expressing cells: ^*^p < 0.05, ^**^p < 0.01, ^***^p < 0.001.

We evoked APs by somatic current injection and recorded fluorescent changes by line-scan imaging at apical dendritic segments (<30 μm from the base). Responses to 1, 2, 5, 10, and 20 APs at 20 Hz and 40 APs at 83 Hz were analyzed (Figures 2, 3 and Table 3). Unexpectedly, we found that none of the GECOs showed any improved performance over GCaMP3. Compared to GCaMP3: G-GECO1.0 was not significantly different over the entire stimulus range tested; G-GECO1.1 showed the same trend, except for smaller responses to 40 APs; G-GECO1.2 showed smaller responses over the entire stimulus range tested except for 1 AP and 40 APs; R-GECO1 showed smaller responses over the entire stimulus range tested except for 1 AP; and GEM-GECO1 showed significantly smaller responses over the entire stimulus range tested. Furthermore, G-GECOs showed slightly yet significantly slower half rise time (Figure 4A; GCaMP3, 236 ± 41 ms, *n* = 13; G-GECO1.0, 280 ± 106 ms, *n* = 9, *p* < 0.05; G-GECO1.1, 282 ± 30 ms, *n* = 10, *p* < 0.01; G-GECO1.2, 257 ± 28 ms, *n* = 9, *p* > 0.05; R-GECO1, 213 ± 65 ms, *n* = 7, *p* > 0.05) and half decay time (Figure 4B; GCaMP3, 190 ± 37 ms, *n* = 13; G-GECO1.0, 260 ± 59 ms, *n* = 9, *p* < 0.05; G-GECO1.1, 301 ± 85 ms, *n* = 10, *p* < 0.001; G-GECO1.2, 249 ± 83 ms, *n* = 9, *p* < 0.05; R-GECO1, 228 ± 73 ms, *n* = 7, *p* > 0.05).

**Table 3:** **SNR of GCaMP3 and GECOs in cortical layer 2/3 pyramidal cells**.

**Number of APs**	**GC3 (*n* = 13)**	**1.0 (*n* = 10)**	**1.1 (*n* = 10)**	**1.2 (*n* = 9)**	**R (*n* = 9)**	**GEM (*n* = 9)**
1	1.5 ± 0.8	1.4 ± 0.2	1.2 ± 0.4	1.1 ± 0.2	1.4 ± 0.9	0.9 ± 0.1^**^
2	2.5 ± 1.6	2.4 ± 1.6	1.7 ± 0.5	1.4 ± 0.4^*^	1.6 ± 0.9^*^	1.0 ± 0.2^***^
5	7.2 ± 6.0	4.8 ± 3.2	4.1 ± 1.8	2.8 ± 1.2^*^	2.4 ± 1.1^**^	1.4 ± 0.3^***^
10	15 ± 12	8.3 ± 5.5	9.0 ± 4.9	5.8 ± 2.9^*^	3.2 ± 1.2^***^	2.1 ± 0.8^***^
20	27 ± 22	15 ± 12	17 ± 9.2	10 ± 6.5^*^	4.8 ± 2.1^***^	3.4 ± 1.0^***^
40	157 ± 39	103 ± 70	59 ± 12^*^	73 ± 39	11 ± 3.1^***^	11 ± 3.2^***^

Action potentials (APs) were evoked at 20 Hz except for 40 APs evoked at 83 Hz. The numbers of recorded cells in the table apply to 1–20 APs; for 40 APs, n = 8 for GCaMP3 and R-GECO1, n = 7 for G-GECO1.0, and n = 9 for G-GECO1.1, G-GECO1.2 and GEM-GECO1. Significant difference in Dunn's post-hoc test comparing GCaMP3-expressing cells and GECO-expressing cells:

* *p* < 0.05,

** *p* < 0.01,

*** *p* < 0.001.

### Comparison with OGB-1

In order to clarify the factors to be improved in the next generation of GCaMP, we quantified the performance of OGB-1, one of the most commonly used synthetic dyes for *in vivo* imaging. We loaded OGB-1 through recording patch pipettes at 20 μM, which is close to the concentration obtained by the bolus loading technique (Stosiek et al., 2003). Consistent with previous studies (Waters et al., 2003; Kerr et al., 2005), OGB-1 reliably detected single APs (Figures 2, 3 and Table 4). Responses of OGB-1 to 1, 2, and 5 APs at 20 Hz were significantly larger than those of GCaMP3. OGB-1 responses showed saturation in response to large number of APs (10 and 20 APs; Figure 2B) as described previously (Yasuda et al., 2004). Responses of OGB-1 to 40 APs were indeed significantly smaller than those of GCaMP3 (Figure 3A and Table 4). Half rise time of OGB-1 was significantly faster than that of GCaMP3 (OGB-1, 26 ± 16 ms, *n* = 8; GCaMP3, 236 ± 41 ms, *n* = 13; *p* < 0.001, Mann–Whitney U test), while half decay time was not significantly different (OGB-1, 215 ± 108 ms, *n* = 8; GCaMP3, 190 ± 37 ms, *n* = 13; *p* = 0.86, Mann–Whitney U test).

**Table 4:** **SNR of OGB-1 in cortical layer 2/3 pyramidal cells**.

**Number of APs**	**OGB-1 (*n* = 8)**
1	6.7 ± 2.1^***^
2	8.7 ± 2.4^***^
5	10 ± 2.8^*^
10	12 ± 3.3
20	13 ± 3.8
40	17 ± 4.3^***^

Action potentials (APs) were evoked at 20 Hz except for 40 APs evoked at 83 Hz. Statistical difference between OGB-1 and GCaMP3 was assessed by Mann–Whitney U test:

* *p* < 0.05,

*** *p* < 0.001.

## Discussion

In the present study, we found that the latest variants of GCaMP or GECOs did not exhibit any improved responses over their ancestor GCaMP3: G-GECOs had lower baseline fluorescence, similar or smaller dynamic range, and slower rise and decay kinetics; R-GECO1 showed expression invading the nucleus and punctate patterns in the cytosol, and had much smaller dynamic range; GEM-GECO1 also had much smaller dynamic range.

### Validity of the techniques used in the present study

The present study was designed to be optimal for efficient comparison across many GECI constructs as well as to be extendable to future *in vivo* experiments.

Ideally, the characterization of all the GECIs should be performed *in vivo*, yet this is not practical for comparison across many constructs. We believe that characterization of GECIs in acute brain slices at physiological temperature should be a reasonable compromise, as it not only gives a good yield of data but also seems to predict *in vivo* GECI performance more reliably than other *in vitro* preparations (Tian et al., 2009; also see the next section).

Compared to other techniques applicable for acute brain slice preparation (virus and transgenic animals), *in utero* electroporation should be preferable for early screening of many constructs, as it is faster and less laborious, only requiring a purified plasmid and pregnant mice for each new construct. One concern may be that Ca^2+^ buffering by GECI expression during the development might perturb the properties of neurons, but the electrophysiological parameters of GECI-expressing neurons were overall not significantly different from those of wild-type cells. It would be interesting to test in the future whether (1) the functionality of GECIs remain comparable when they are expressed for a longer period of time (Mank et al., 2008; Tian et al., 2009; Yamada et al., 2011) and (2) different transfection methods (virus and transgenic animals) can result in different GECI performance, especially when more promising GECIs are developed and validated.

Imaging in this study was performed exclusively at the proximal apical dendritic segments as previously described (Pologruto et al., 2004; Mao et al., 2008; Tian et al., 2009; Yamada et al., 2011), yet the future experiments should be preferably performed at the soma, where most *in vivo* multi-cell imaging are performed. Since AP-associated Ca^2+^ transients are generally smaller in the soma than in the proximal apical dendrites (Schiller et al., 1995), the apparent performance of GECIs would be expected to be lower, presumably making the criteria for GECI selection even more stringent.

### Comparison with previous studies using the same GECIs

Like many other variants of GCaMP generated by other groups (Souslova et al., 2007; Muto et al., 2011), the relationship between fluorescent responses of GECOs and the number of APs was poorly characterized in the original study, where it was claimed that GECOs showed larger dynamic range compared to GCaMP3 (Zhao et al., 2011). The source of inconsistencies between the original results and ours is currently unknown, yet they might be attributed to the difference in preparation (culture in the original study vs. acute slice in ours) as well as whether or not imaging was accompanied with electrophysiology. Given that GCaMP3 showed much larger single AP responses in culture (Δ*F*/*F*_0_: 46 ± 4.2%) than in acute slice preparation (14 ± 2.7%) or *in vivo* (7.9 ± 2.8%) (Tian et al., 2009), we believe that the experimental design used here should be better suited to assess the actual performance of GECIs in mammalian neurons.

### Implications for future improvement of GCaMP and other GECIs

As indicated from the comparison with OGB-1, one of the obvious goals for the next generation of GCaMP is reliable detection of single APs. For successful improvement, appropriate guidelines for evaluating the efficacy of GECIs should be established and accepted in the research community (Hires et al., 2008). We propose that the following implications drawn from our study should be taken into consideration: (1) Properties of GECIs (dynamic range, affinity, kinetics, etc.) measured with purified protein often show limited correlation with the performance in neurons (e.g., Purified protein of GEM-GECO1 has a larger dynamic range and a higher affinity compared to GCaMP3, but performs far worse in neurons; see similar reports in Hendel et al., 2008); (2) Screening of optimal GECIs for mammalian neurons should be finalized with combined imaging and electrophysiology in a non-culture system; and (3) GECI performance can be strikingly different from one species to another (e.g., GEM-GECO1 showed limited responsiveness in mouse cortical neurons, but good functionality in *C. elegans* sensory neurons).

We also propose that factors responsible for the reduced GECI performance in mammalian neurons in physiological preparations should be identified and overcome; these might include interaction of GECIs with endogenous CaM (Miyawaki et al., 1999; Palmer et al., 2006) or with other as yet unknown binding proteins, or direct modifications of GECI protein such as phosphorylation. Some useful clues might be obtained by biochemical comparison of cell lysates containing GECI protein from different cell types (e.g., HeLa cells, where GECOs show high performance vs. cortical pyramidal cells, where GECOs show reduced performance) or from the same type of neurons in different preparations.

We hope that our findings will alert the research community to the limitations of current GECIs, stimulate future development and screening of a “holy-grail” GECI with high sensitivity and fast kinetics, and facilitate appropriate selection of optimal GECIs for different experimental requirements.

### Conflict of interest statement

The authors declare that the research was conducted in the absence of any commercial or financial relationships that could be construed as a potential conflict of interest.
